# The Optimization of a Protocol for the Directed Differentiation of Induced Pluripotent Stem Cells into Liver Progenitor Cells and the Delivery of Transgenes

**DOI:** 10.3390/biology14060586

**Published:** 2025-05-22

**Authors:** Irina Panchuk, Valeriia Kovalskaia, Natalia Balinova, Oxana Ryzhkova, Svetlana Smirnikhina

**Affiliations:** Research Centre for Medical Genetics, Moskvorechye Str., 1, 115478 Moscow, Russia; mikhailova.v.a@mail.ru (V.K.); balinovanatasha@gmail.com (N.B.); ryzhkova@dnalab.ru (O.R.); smirnikhinas@gmail.com (S.S.)

**Keywords:** induced pluripotent stem cells (iPSCs), directed differentiation, liver progenitors, 3D organoids, recombinant adeno-associated viral vector, transfection

## Abstract

The liver plays a pivotal role in metabolism, detoxification, and protein synthesis and comprises several cell types, including hepatocytes and cholangiocytes. Primary human hepatocytes in 2D cultures rapidly dedifferentiate and lose their function, making their use as a reliable cell model challenging. Therefore, developing robust three-dimensional cell culture models is crucial, especially for diseases lacking reliable animal models. The aim of this study was to optimize a protocol for the directed differentiation of induced pluripotent stem cells into liver progenitor cells, achieving the high-level expression of specific markers. As a result, we established a 2D culture of liver progenitor cells capable of differentiating into three cell types: a 3D organoid culture containing hepatocyte- and cholangiocyte-like cells and a 2D cell culture comprising stellate-like cells. To evaluate gene delivery efficiency, liver progenitor cells were transduced with various rAAV serotypes carrying an eGFP reporter cassette at different multiplicities of infection (MOIs). Our results revealed that rAAV serotype 2/2 at MOI of 100,000 achieved the highest transduction efficiency of 93.6%, while electroporation demonstrated a plasmid delivery efficiency of 54.3%. These findings suggest that liver progenitor cells are a promising tissue-like cell model for regenerative medicine and demonstrate high amenability to genetic manipulation, underscoring their potential in gene therapy and genome editing studies.

## Simple Summary

The liver is essential for detoxification, metabolism, and protein synthesis but is at risk of serious diseases, such as fatty liver disease, hepatitis, and liver cancer. Cell-based models are critical for advancing research and developing effective therapies. However, primary human hepatocytes are challenging to use for disease modeling due to their dedifferentiation and loss of functional properties in vitro. Additionally, animal models are often non-relevant for certain liver diseases. To address these limitations, 3D cell culture models, such as organoids, have emerged as promising tools for studying liver physiology and disease progression. Recent studies have focused on optimizing protocols to differentiate induced pluripotent stem cells (iPSCs) into liver progenitor cells (LPCs). During directed differentiation, cells undergo stages that closely resemble those observed during the embryonic development of liver tissue. We optimized a protocol to generate cultures both in 2D and 3D, achieving high differentiation efficiency for key hepatocyte markers. We also evaluated two transgene delivery methods: viral (recombinant adeno-associated viruses) and non-viral (electroporation), achieving efficiencies of 93.6% and 54.3%, respectively. The resulting cell models can be used for disease modeling, drug screening, and personalized therapies, including genome editing and gene therapy applications.

## 1. Introduction

The liver is a vital organ involved in metabolic processes, such as detoxification, amino acid and lipid metabolism, and serum protein synthesis. It comprises both parenchymal and non-parenchymal cells, such as hepatocytes, cholangiocytes, sinusoidal endothelial cells, Kupffer cells, and stellate cells. Hepatocytes and cholangiocytes derive from a common precursor, hepatic stem cells, during embryonic liver development. Hepatocytes, the primary liver parenchymal cells, are essential for metabolic processes, while cholangiocytes, a type of epithelial cell, regulate bile duct formation and bile acid transport [1,2,3].

Primary human hepatocytes are limited as experimental models due to rapid dedifferentiation in 2D cultures, particularly in the absence of a tissue-specific extracellular matrix. In addition, the lack of reliable animal models for several liver diseases, such as autoimmune hepatitis, primary biliary cholangitis, and non-alcoholic fatty liver disease/non-alcoholic steatohepatitis (NAFLD/NASH), limits research progress. Thus, developing 3D cell culture models that reproduce liver physiology and cellular characteristics is critical for in vitro studies of pathogenesis and the development of therapeutic strategies.

In the early 2000s, some of the first articles on liver cell models were published and indexed in the PubMed database. These papers focused on embryonic mice and rat cells and their potential to differentiate into hepatocyte-like cells. Between 2010 and 2018, the majority of studies aimed to differentiate induced pluripotent stem cells (iPSCs) into hepatocyte-like cells and optimize related protocols [4]. Over the last five years, research has focused on the development of heterogeneous cultures and three-dimensional cell models, including organoids and microfluidic models [5]. Cellular models based on human iPSCs (hiPSCs) have become a valuable tool in biomedical research and drug discovery. These models are created by reprogramming somatic cells into an embryonic-like state, followed by spontaneous or directed differentiation into specific cell types. Liver progenitor cells (LPCs), a bipotent, tissue-specific cell population, are capable of self-renewal and differentiation into specialized liver cells. These cells have a wide range of applications in 2D and 3D cell culture, including disease modeling, pathogenesis studies, drug development and testing, and the gene editing of pathogenic variants.

Organoid technology, a form of 3D cell culture, is essential for studying the structure, function, and molecular and cellular characteristics of tissues and organs. Organoids are used to model disease, to understand their mechanisms, and to identify potential therapeutic targets [6,7]. These models enable the study of disease progression, drug efficacy, and candidate drug discovery in a more physiologically relevant system than traditional cell lines or animal models. This approach offers the promise of precision medicine, enabling personalized therapies based on genetic and disease profiles [8]. Research aims to increase the expression of cytochrome P450 enzymes, such as CYP3A4 and CYP1A2, which are crucial for drug metabolism. Overall, liver organoids represent a significant advance in biomedicine, supporting disease modeling [9,10,11], precision medicine [12,13], and drug development [14], although achieving full physiological functions and maturation remains a challenge [5,11,15,16].

The aim of this study was to optimize a protocol for the directed differentiation of hiPSCs into LPCs that is rapid, cost-effective, and straightforward. A key aspect was to standardize the concentrations of small molecules and growth factors, thereby reducing the need for the individualized optimization for each cell line. This standardized approach is expected to improve the reproducibility of experiments, reduce costs, and contribute to more efficient and scalable scientific progress in the field [17]. The developed 2D LPCs model will be used for transgene delivery using viral and non-viral methods. This task is particularly relevant given the limited number of published studies dedicated to this topic.

## 2. Materials and Methods

### 2.1. Obtained hiPSC’s Cell Line

Skin fibroblasts from three patients (P10L1, P17L16, and P11L3) and two healthy donors (P16L4 and P12L3) were reprogrammed into hiPSCs using the CytoTuneTM-iPS 2.0 Sendai Reprogramming Kit (Thermo Fisher Scientific, Waltham, MA, USA), according to the manufacturer’s instructions. The resulting hiPSC lines were seeded onto Matrigel-coated plates (Corning, NY, USA) and cultured in a TeSR-E8 medium (STEMCELL Technologies, Vancouver, BC, Canada) with daily medium changes to maintain their growth and pluripotency. The cell lines were characterized for the presence of the expression of pluripotency markers by immunofluorescence analysis with antibodies against SSEA (Thermo Fisher Scientific, USA), NANOG (Thermo Fisher Scientific, USA), OCT-4 (Abcam, Cambridge, UK) and by directed differentiation to achieve ectodermal (beta III tubulin (Abcam, UK)), mesodermal (brachyury (eBioscience, San Diego, CA, USA)), and endodermal (FOXA2 (Abcam, UK)) markers. The cell lines were found to be mycoplasma-free [18,19,20]. The characterization of P12L3 is presented in the Supplementary Materials.

### 2.2. Directed Differentiation

**Figure 1:**
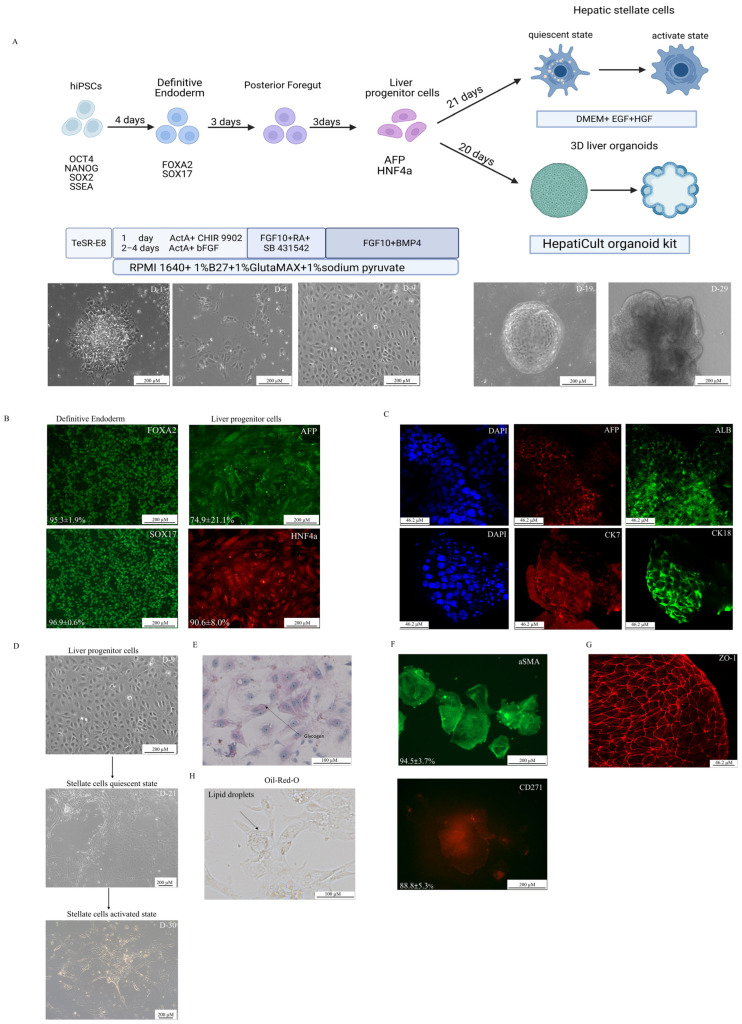
**Differentiation of hiPSCs into liver progenitor cells (LPCs) and stellate-like cells.** (**A**) A schematic of the differentiation protocol with phase-contrast microscopy images showing morphological changes at key stages of differentiation (D = day). Scale bars: 200 µm. (**B**) An immunofluorescence analysis of definitive endoderm (DE) and LPC markers during 2D differentiation. Scale bars 200 µm. (**C**) 3D culture immunofluorescence of differentiated LPCs. Scale bars: 46.2 µm. (**D**) Differentiation scheme of LPCs into hepatic stellate cells. Scale bars: 200 µm. (**E**) Periodic acid–Schiff (PAS) staining demonstrating glycogen storage in LPCs. Scale bars: 100 µm. (**F**) Immunofluorescence of 2D, cultured stellate cells. Scale bars: 200 µm. (**G**) Tight junction formation in 3D cultures visualized by ZO—1 immunofluorescence. Scale bars: 46.2 µm. (**H**) Positive Oil-Red-O staining demonstrated intracellular lipid storage in hepatic stellate cells. Scale bars: 100 µm.

The hiPSCs were harvested using a Versen solution (PanEco, Moscow, Russia) and seeded at a density of 100,000 cells per cm^2^ in a 12-well plate (SPL Life Science, Pocheon-si, South Korea) pre-coated with Matrigel. The hiPSCs were sequentially differentiated into definitive endoderm (DE) cells using a basal medium (RPMI 1640 Medium (STEMCELL Technologies, Canada), 1% B-27 supplement without Vitamin A (Thermo Fisher Scientific, USA), 1% Glutamax (Thermo Fisher Scientific, USA), 1% sodium pyruvate (PanEco, Russia)), 100 ng/mL Activin A (STEMCELL Technologies, Canada), and 3 µM CHIR99021 (Tocris, Bristol, UK) for the first 24 h, then 100 ng/mL Activin A and 10 ng/mL FGF_β_ (PanEco, Russia) were added for the next three days. The anteroposterior foregut was cultured in a basal medium supplemented with 50 ng/mL FGF10 (R&D System, Minneapolis, MN, USA), 10 µM SB431542 (Tocris, UK), and 10 uM retinoic acid (Sigma Aldrich, St. Louis, MO, USA). Liver progenitor cells (LPCs) were cultured in a basal medium supplemented with 50 ng/mL FGF10 and 10 uM BMP4 (R&D System, USA). The media were changed daily. The HepatiCult Organoid Kit (STEMCELL Technologies, Canada) was used to initiate the growth and differentiation of 3D liver organoids according to the manufacturer’s instructions. For the generation of 3D organoids, cells were harvested with Versen, counted, and the suspension was centrifuged at 1000× *g* for 5 min. After removing the supernatant, 20 µL of a Matrigel stock solution was added per 20,000 cells. Organoid droplets were formed in 12-well plates and incubated for 40–60 min at 37 °C before the medium was added. A conditioned medium based on the HepG2 cell line was prepared to obtain liver stellate cells. The medium consisted of DMEM (PanEco, Russia), 10% FBS (BioSera, Cholet France), and 1% Glutamax, supplemented with 20 ng/mL EGF (R&D System, USA) and 20 ng/mL HGF (ABclonal Technology, Woburn, MA, USA) (Figure 1A). The conditioned medium was collected from a T75 flask with a total volume of 13 mL, centrifuged at 1000× *g* for 5 min to remove cellular debris, and the supernatant was filtered using a 0.66 µm membrane filter. Finally, the medium was aliquoted and stored at −20 °C. The medium was changed every other day. Appropriate differentiation stages were confirmed by evaluating the expression of markers characteristic for each stage using immunocytochemistry, flow cytometry, and analysis with Cell Profiler 3.0.0 software. The cells were incubated at 37 °C, 5% CO_2_, and >90% humidity.

### 2.3. Immunocytochemistry

The cells were fixed using 3.7% paraformaldehyde (Carl Roth, Karlsruhe, Germany) for 20 min at room temperature (RT), then permeabilized with 0.25% Triton X-100 (Servicebio Technology, Wuhan, China) and blocked with 1% bovine serum albumin (BSA) (Sigma Aldrich, USA). Subsequently, the primary and secondary antibodies were incubated for one hour in the dark and 30 min at room temperature, respectively. The list of antibodies used is presented in Table 1. The nuclei were then stained with DAPI (Abcam, Cambridge, UK) for 10 min at room temperature. Fluorescent images were captured using the Lionheart FX Automated Microscope (BioTek, Shoreline, WA, USA), and the number of positive cells was analyzed using the open-source software CellProfiler 3.0.0. The macros and image processing used for the segmentation analysis are provided in the Supplementary File «*.cpproj».

### 2.4. Oil-Red-O Staining

The staining procedure was performed according to the manufacturer’s instructions, using the EZstain Adipocyte Staining Kit (HIMEDIA, Mumbai, India). Briefly, the procedure consisted of washing the cells, followed by fixation and permeabilization. The working solution for staining was then prepared by mixing three parts of Oil-Red-O staining solution with two parts of distilled water. The samples were incubated at room temperature for ten minutes, followed by the photo-detection of the staining.

### 2.5. Non-Viral and Viral Transgene Delivery

The Neon Electroporation System (Thermo Fisher Scientific, USA) with 10 µL Neon tips was used for electroporation of 30,000 LPCs (1 well of a 12-well plate) with an AAV-CMV-GFP (2 µg) plasmid, which was a gift from Connie Cepko (Addgene plasmid # 67634; http://www.addgene.org/67634/ (accessed on 10 January 2025); RRID:Addgene_67634), and dsCFTR (1 µg), which was a gift from Tobias Cantz (AAT-PB-CG2APtk, http://www.addgene.org/86003 (accessed on 10 January 2025), RRID:Addgene_86003). A cell culture after Neon electroporation without a plasmid was used as a negative control. Transfection was performed according to the recommendations of the Neon transfection protocol. The transfection efficiency was evaluated by flow cytometry (CytoFLEX, Beckman Coulter, Brea, CA, USA) at 48 h.

The methodology for the production and purification of recombinant adeno-associated viral vectors (rAAVs) was described previously [21]. A total of 30,000 LPCs were seeded onto a 12-well plate 24 h before transduction. One hour before transduction, the medium was changed to a fresh solution. For transduction, rAAV serotypes 2/2, 2/5, 2/6, and 2/9, with a GFP expression cassette were used at an MOI (multiplicity of infection) of 10,000 and 100,000. Transduction efficiency was evaluated at 72 h by flow cytometry (CytoFLEX, Beckman Coulter, USA). Data were obtained in two technical replicates.

### 2.6. Statistical Analysis

Statistical analysis was performed using GraphPad Prism v.9.1.1 software. A t-test was used to compare the percentage of positive cells stained by antibodies. The Shapiro–Wilk test followed by the Tukey’s post hoc method was used to evaluate the efficiency of transduction and transfection. Data were considered statistically significant if the *p*-value was below 0.05.

### 2.7. Gene Expression Analysis

Human Liver Total RNA (AM7960, Invitrogen, Carlsbad, CA, USA) was used as a gene expression control. 3D organoids cultured for 29 days were manually harvested in a DMEM medium (PanEco, Russia) supplemented with 5% FBS (BioSera, France). The samples were centrifuged at 6100× *g* for 2 min, and the supernatant was removed. RNA was isolated using a TRIzol reagent (Invitrogen, USA). The RNA concentration was measured spectrophotometrically using a Nano-500 (Allsheng, Hangzhou, China), and the RNA quality was assessed via 1% agarose gel electrophoresis and capillary electrophoresis by TapeStation 4200 (Agilent, Lexington, MA, USA). Only samples with an RNA concentration of >50 ng/µL, 260/280 ≈ 2, 260/230 > 2, and an RNA Integrity Number (RIN) of >6 were selected for RNA seq. Ribosomal RNA (rRNA) was depleted using the Ribo-off rRNA Depletion Kit (Vazyme, Nanjing, China). RNA libraries were prepared using the MGIEasy RNA Library Prep Kit (MGI, Wuhan, China) following the manufacturer’s instructions. The concentration of the libraries was subsequently determined using a Qubit 2.0 fluorometer (Invitrogen, MA, USA). The library size distribution and quality were then assessed using a TapeStation DNA ScreenTape instrument (Agilent, USA).

Prior to cyclization, coding sequences corresponding to approximately 20,000 genes were enriched using the NEXome Plus Panel v1.0 (NEXome Core, Nanodigmbio, Nanjing, China). Cyclization was performed using the MGIEasy Cyclization Module (MGI, China) in accordance with the manufacturer’s guidelines. Subsequently, the DNBSEQ-G400 (BGI, Shenzhen, China) platform was utilized for the purpose of sequencing, employing the DNBSEQ-G400 high-throughput sequencing kit, generating 70–100 million reads per sample.

Raw sequencing reads were processed using an NGS analysis pipeline developed at the Research Centre for Medical Genetics (RCMG), which integrates established tools with custom optimizations for RNA-seq data [22]. For the differential expression analysis, raw counts were processed using DESeq2; genes with <10 average reads across all the samples were excluded.

## 3. Results

### 3.1. Directed Differentiation

The directed differentiation protocol was optimized using five iPSC lines. Definitive endoderm differentiation was confirmed by the expression of SOX17 (96.9 ± 0.6%) and FOXA2 (95.3 ± 1.9%) markers (Figure 1B). The presence of LPCs was validated by the co-expression of AFP and HNF4α (74.9 ± 21.1%) (Figure 1B) and positive staining for glycogen storage (Figure 1E). In the next step, two different cultures were established: a 3D cell culture of hepatic organoids (Supplementary Figures S1 and S2) and a 2D cell culture of stellate-like cells. During the 3D differentiation process, hepatic organoids showed the positive expression of hepatocyte-specific markers (CK18+, ALB+, AFP+) and cholangiocyte-like cells (CK7+). The organoids were also stained for the ZO-1 marker to demonstrate the 3D cell culture organization (Figure 1C,G, Supplementary Figure S1). Hepatic stellate cells derived from LPCs were confirmed by the Oil-Red-O staining of lipid droplets (Figure 1H) and the presence of specific morphology. (Supplementary Materials Video S1) Further characterization revealed the high expression of stellate cell markers, as well as CD271 (88.8 ± 5.3%) and a-SMA (94.5 ± 3.7%) (Figure 1F). Furthermore, liver stellate cells were derived from LPCs in both quiescent and activated states after transition into myofibroblasts, with microscopy evidence of cellular contraction (Supplementary Materials Video S1). The LPC cultures were successfully obtained and subsequently differentiated into hepatocyte- and cholangiocyte-like cells.

Both the 2D and 3D hepatic cell cultures demonstrated the expression of key hepatocyte-associated markers, including AFP (a fetal liver marker), HNF4α (a key transcriptional regulator of hepatic differentiation), and EpCAM (a marker of hepatic progenitors and cholangiocytes). Transcriptomic profiling using the ADME Core Panel (drug absorption, distribution, metabolism, and excretion) revealed the significant downregulation of crucial phase I drug-metabolizing enzymes (*CYP3A4*, *CYP2C9*, *CYP2C19*, *CYP1A2*, *CYP2C8*, *CYP2B6*, *CYP2E1*) and transporters (*SLCO1B1*, *SLCO2B1*, *UGT1B15*, *SLC22A1)* in 3D liver organoids compared relative to native human liver tissue. The organoids showed the elevated expression of immature (fetal) hepatic markers (*CYP3A7*, *CYP1A1*, *AFP*) while maintaining near-physiological expression levels of certain phase II detoxification enzymes (*GSTP1*, *GSTT2*, *SULT1A1*, *NAT1*) and selected transporters (*ABCB1*, *ABCC2*, *ABCG2*, *SLC22A6*, *SLC15A2*, *SLC22A2*). The organoid’s expression profile exhibits an immature liver phenotype (*CYP3A7* high, *AFP* high, *CYP3A4* low, *ALB* low) with partially preserved detoxification capacity (*GSTP1* high, *SULT1A1* retained). This phenotypic profile makes the organoids particularly suitable for studying hepatic development.

### 3.2. Non-Viral Transfection

Liver progenitor cells are valuable models for studying hepatic disorders and hold significant potential for the development of novel therapeutic strategies targeting liver diseases. Gene therapy, including genome editing approaches, is a promising tool for treating human diseases. This underscores the importance of developing efficient transgene delivery protocols for in vitro studies. In the present work, we optimized the delivery of an AAV-CMV-GFP plasmid by a non-viral method (electroporation) and viral delivery methods, using an rAAV containing the same reporter gene—*eGFP*.

**Figure 2:**
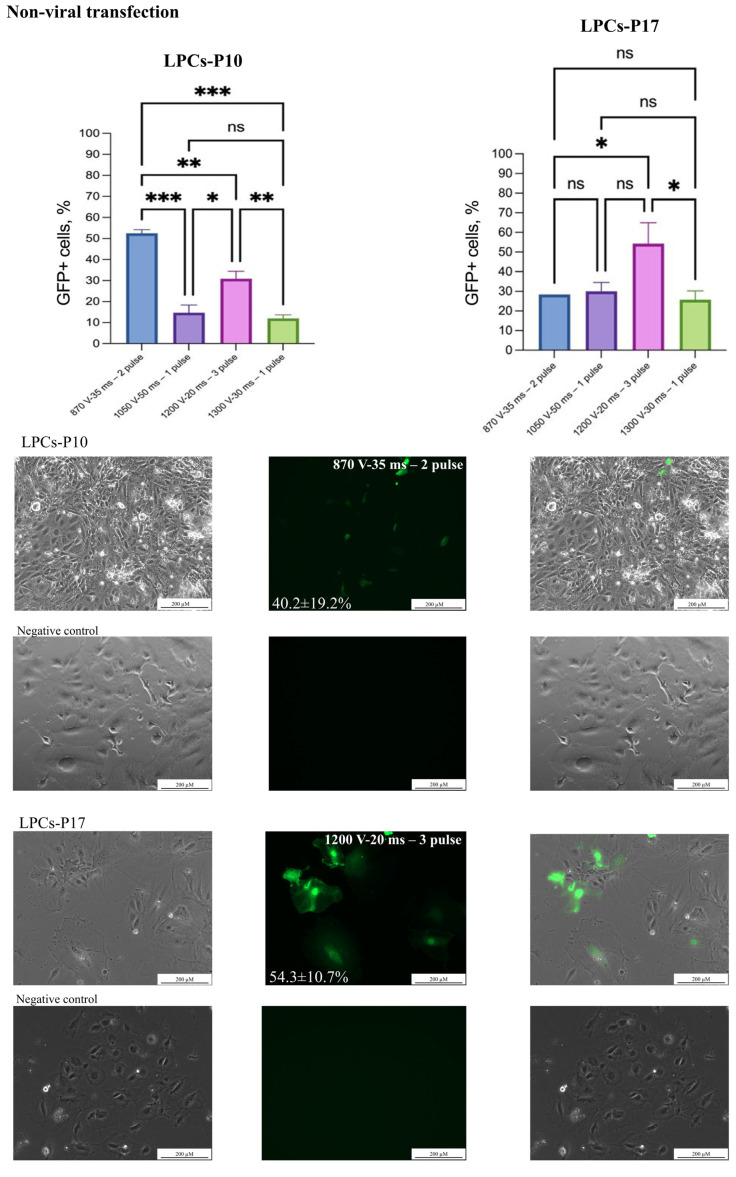
**Non-viral transgene delivery into LPCs**. Two LPC lines were transfected with AAV-CMV-GFP plasmid by electroporation. Scale bars 200 µm. The figure presents the best result of transfection of the LPC lines in the phase-contrast microscopy, GFP-transfected cells, and merged images. The results are presented as mean ± SD, n = 2. The statistical analysis included the Shapiro–Wilk test, followed by Tukey’s post hoc test. Significance levels are indicated as follows: * *p* < 0.05, ** *p* < 0.01, *** *p* < 0.001, ns—not significant.

**Figure 3:**
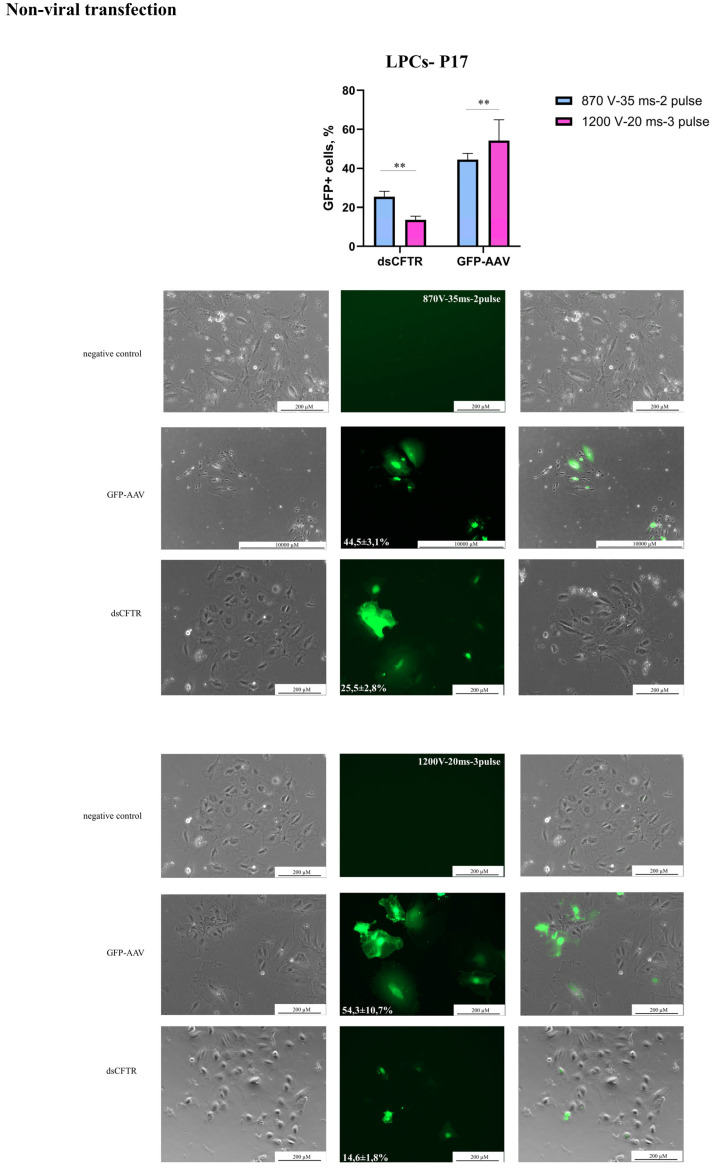
**Non-viral transgene delivery into LPCs-P17**. Line LPCs-P17 was transfected with AAV-CMV-GFP and dsCFTR plasmid by electroporation. Scale bars: 200 µm, 1000 µm. The figure presents the best result of the transfection of the line in the phase-contrast microscopy, GFP-transfected cells, and merged images. The results are presented as mean ± SD, n = 2. The statistical analysis included the Shapiro–Wilk test, followed by Tukey’s post hoc test. Significance levels are indicated as follows: ** *p* < 0.01.

Initially, the electroporation protocols recommended by Neon’s manufacturer for commonly used hepatocyte cell lines (ChangX-31, HepG2, SK-Hep-1, H-4-II-E) were used to develop the transfection protocol (Table 2). A plasmid transfection protocol was established using 2 μg/μL of the AAV-CMV-GFP with 30,000 LPCs and 10 μL Neon tips (Table 2). The 1600 V/20 ms/L pulse protocol caused LPC death and was considered unsuitable. In our experiments, a DNA concentration of 3 μg/μL resulted in significant cell death and the suboptimal efficiency of AAV-CMV-GFP plasmid delivery. This assessment was conducted by microscopy. However, using 2 μg/μL of an AAV-CMV-GFP plasmid significantly improved cell survival and delivery efficiency (Table 2), (Figure 2). In experiments with the dsCFTR plasmid, optimal results were achieved using 1 μg/μL and two distinct electroporation protocols (see Figure 3). However, increasing the plasmid concentration to 2 µg/µL led to elevated cell death. The Neon transfection system provided the most optimal conditions for AAV-CMV-GFP delivery, with transfection efficiencies of 54.3 ± 10.7% for LPCs-P17 and 52.5 ± 1.8% for LPCs-P10 (Figure 2). For dsCFTR plasmid delivery, the efficiency was 25.5 ± 2.8% in LPCs-P17 (Figure 3).

### 3.3. Viral Transduction

**Figure 4:**
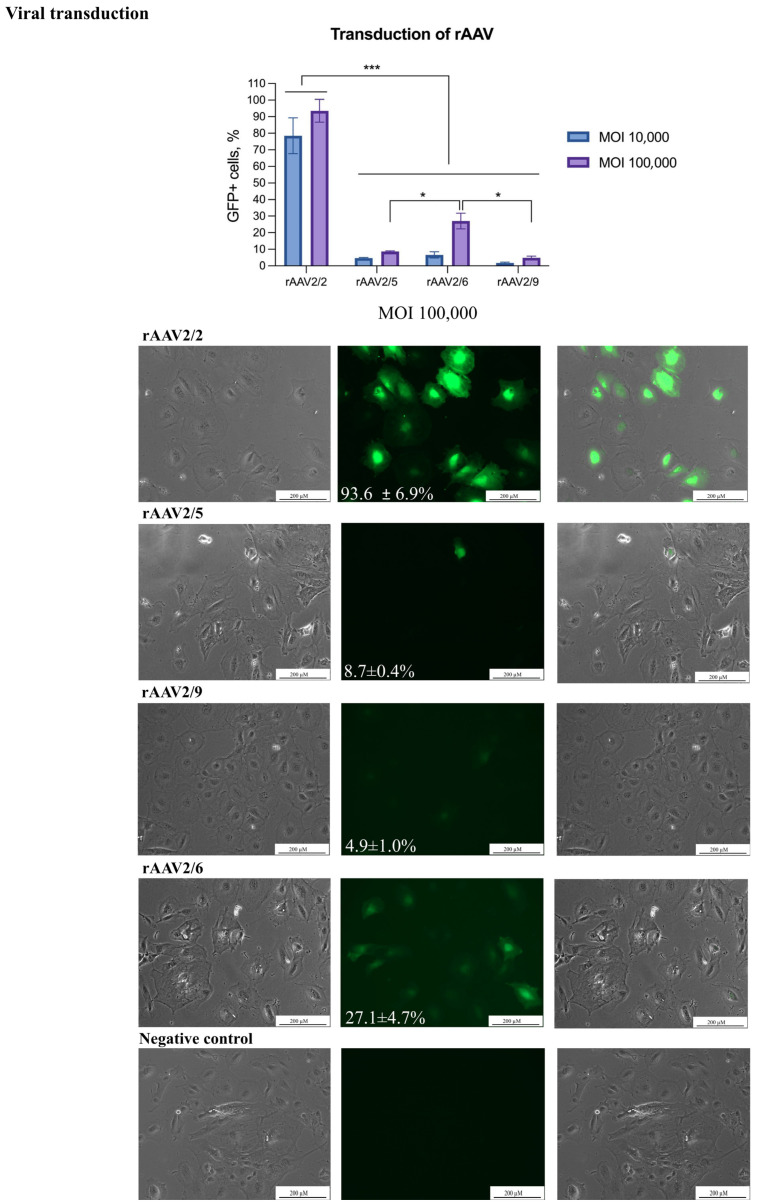
**Transduction of LPCs by rAAVs**. The transduction of the LPCs was performed using rAAVs serotypes 2/2, 2/5, 2/6, and 2/9. Scale bars: 200 µm. The figure presents the transduction of LPCs in the phase-contrast microscopy, GFP-transduced cells, and merged images. The results are presented as mean ± SD, n = 2. The statistical analysis included the Shapiro–Wilk test, followed by Tukey’s post hoc test. Significance levels are indicated as follows: * *p* < 0.05, *** *p* < 0.001.

Liver progenitor cells were transduced by rAAVs serotypes 2/2, 2/5, 2/6, and 2/9 with an *eGFP* reporter cassette at MOIs of 10,000 and 100,000. The best results were obtained using an rAAV serotype 2 at an MOI of 100,000 with a transduction efficiency of 93.6 ± 6.9% (Figure 4). In contrast, serotypes 2/5 and 2/9 showed efficiencies below 10%, even at the highest viral doses. Serotype 2/6 demonstrated a reporter gene delivery efficiency of 27% at the highest tested MOI tested.

## 4. Discussion

The variety of cellular models for studying hepatic diseases and developing therapies allows for both initial drug testing and functional studies using 3D structures. In 2021, the FDA published a document exploring the potential use of 3D cell models as an alternative for animal testing in preclinical studies [23] The creation of organoids from both healthy and diseased tissues makes them valuable for disease modeling [15]. The utilization of three-dimensional models derived from human cells has the potential to provide more relevant research findings, reduce harm to animals, and improve cost-efficiency. The availability of different techniques for obtaining 2D and 3D cellular liver models underscores the necessity for the development of straightforward, cost-effective, rapidly reproducible methods. This study aimed to establish a protocol for obtaining liver progenitor cells in both 2D, which facilitate experimental simplicity, and 3D organoids, which more closely imitate the liver’s structural and functional complexity.

We recommend culturing hiPSCs in commercial media, such as mTeSR™1 or TeSR™-E8™ (both from STEMCELL Technologies, Canada), on Matrigel-coated plates (Corning, USA). Under these conditions, hiPSCs maintain their characteristic morphology and high proliferative potential. The effectiveness of the differentiation process depends on the quality of the pluripotent stem cells, their initial seeding density, proliferation rate, and the culture conditions [24,25]. Consequently, we do not recommend using cell cultures containing more than 10% of cells with atypical hiPSC morphology, as this may adversely affect the outcome. For the subculturing of hiPSCs, gentle enzymatic reagents, such as Versen or StemPro^®^ Accutase^®^ Cell Dissociation Reagent (Thermo Fisher Scientific, USA), are preferable [26]. Protocols for the directed differentiation of hiPSCs into hiPSC-derived liver cells follow a standardized process involving several steps: endoderm induction, liver initiation, liver specification, and liver maturation. The basal media may include DMEM/F12, DMEM, RPMI 1640, L-15, IMDM, and B27, with or without vitamin A or insulin. In our protocol, Glutamax and sodium pyruvate are also included to support proliferation and provide a suitable carbohydrate source to maintain the glycolysis–TCA cycle and ATP production [2,27]. For endoderm induction, the STEMdiff Definitive Endoderm Differentiation Kit (STEMCELL Technologies) or Activin A or Wnt in combination with other reagents are commonly used. This stage represents the most critical phase of the differentiation process. The successful evaluation of DE markers FOXA2 and SOX17 is crucial. FOXA2 is essential for hepatocyte development and suppression cholangiocyte proliferation, while SOX17 expression is crucial for definitive endoderm formation [24,28]. To induce the primitive streak stage, most protocols recommend the addition of 100 ng/mL Activin A and 3 µM CHIR99201 to the basal medium on the first day of differentiation. Following this, 100 ng/mL Activin A and 10 ng/mL FGFb are added for 2–3 days to achieve the definitive endoderm (DE) stage. The quantitative analysis shows that SOX17/FOXA2 levels exceed 90% [1], along with gene expression of *SOX17*, *FOXA2*, and *GATA4* [29]. The differentiation stage of hepatic progenitor cells is commonly achieved using a variety of media, including DMEM/F12, F12, DMEM, and knockout DMEM. In our work, we use a single basal medium for all the 2D differentiation steps, making our protocol simpler and more cost-effective. The small molecules and growth factors used at hepatic progenitor stage vary notably across the protocols, and comparisons can be made using specific markers. Ao et al. and Gao et al. [2,29] have demonstrated the efficient differentiation of hepatocyte progenitors, achieving an 85.8% AFP detection rate [25], 72.3% AFP and HNF4a co-expression [26], and statistically higher *AFP*, *TTR*, and *HNF4a* gene expression [2,8]. Our results show AFP+HNF4a+ at 74.9%. The liver maturation stage is usually performed using commercial kits (William’s E Medium with Primary Hepatocyte Maintenance Kit or the HepatiCult organoid kit), which complicates the cross-study comparison of results. The differentiation of hepatic cells is evaluated by the expression of *HNF4a*, *AFP*, *ALB,* and *CYP3A4*, as well as the qualitative and quantitative analysis of immunocytochemistry results and functional assays (urea and glycogen storage) [1,29,30,31,32,33].

The quantitative data on marker expression obtained in this study are consistent with previously published results. A comparison of the present approach with other protocols (see Table 3) demonstrates that it achieves high efficiency in differentiating definitive endoderm cells while requiring fewer small molecules and growth factors [25,34].

Hepatic stellate cells in the liver store vitamin A in lipid droplets and produce an extracellular matrix, regulate sinusoidal blood flow, and contribute to liver fibrosis. Recent research has revealed their role in liver inflammation by producing inflammatory molecules and interacting with other liver cells. Hepatic stellate cells have the capacity to either enhance inflammation or suppress damage through immunoregulatory signaling. Further studies are required to understand their full range of functions and potential therapeutic applications in inflammatory conditions in various organs. Liver injury activates hepatic stellate cells, resulting in the loss of their lipid storage function, a change in their phenotype, and their subsequent transformation into myofibroblasts. These myofibroblasts (aSMA+) produce increased amounts of extracellular matrices, proinflammatory molecules, and substances that promote scarring, leading to liver fibrosis [35]. Hepatic stellate cells are typically derived from mesodermal precursors [3,34,36,37]; however, our study suggests an endodermal origin of these cells. The origin of stellate cells remains a controversial issue. Notably, SOX17+ mouse DE cells can further differentiate into definitive endoderm-derived mesenchymal cells [38], supporting this hypothesis. In the present protocol, the duration of the differentiation process is 30 days to obtain this cell type (versus 10–14 days in other studies). The present protocol has demonstrated the generation of three different cell types from a single precursor: hepatocyte-like, cholangiocyte-like, and stellate-like cells. Additionally, this approach facilitates the generation of heterogeneous cell cultures, which more closely replicate the characteristics observed in in vivo studies.

The delivery of transgenes into cells is a process with a variety of applications in research and biotechnology, including the study of gene function, gene expression studies, and the development of gene therapies. AAV transduction is widely recognized for its ability to effectively deliver genetic material into a wide range of cell types, including both dividing and non-dividing cells, as well as traditionally challenging-to-transfect cells. It is important to understand the capacity of AAV vectors to package genetic material, which is typically limited to 4.7–4.9 kb. This limitation can constrain the size of the transgene that can be efficiently delivered [11]. For larger DNA constructs, electroporation (e.g., the Neon transfection system) is a suitable method but may cause cellular stress and toxicity, especially in sensitive cell types [39]. AAV transduction is considered a less harmful and gentler method for delivering transgenes into cells. Conversely, electroporation can cause cellular stress and toxicity, especially in sensitive cell types. The choice of transgene delivery method (AAV transduction or Neon transfection) depends on the specific experiment or application requirements, such as the cell type, transgene size, desired expression duration, and potential effects on cell viability and genomic stability. The optimization of the transgene delivery protocol is essential to balance delivery efficiency and cell survival [25].

The transfection of primary and cancerous liver cell lines, including primary human hepatocytes (PHH) and HepG2, presents significant challenges [25]. A range of lipofectamine-based methods, including Lipofectamine 2000, Metafectene, and Targefect, were tested to optimize transfection conditions in primary hepatocytes. The best results were observed using Targefect with primary mouse hepatocytes (4 × 10^5^ cells) transfected with 2 μg of DNA, yielding an efficiency of ~50.6 ± 1.9%. Additionally, 4.5 μg of siRNA or miRNA resulted in a transfection efficiency of more than 90% after 16 h [8] For alternative approaches, polyethylenimine (PEI) nanoparticles were used with 0.4 μg of DNA per 200–250 cells to transfect both primary mouse hepatocytes and HepG2 cells. The transfection efficiency was measured by relative light units (RLUs), ranging from 10,000 to 100,000 RLUs [8], with the best result being 10,000,000–100,000,000 RLUs [40]. In 2023, a high transfection rate of 47% was achieved in primary Atlantic salmon hepatocytes by the Neon transfection method, using 3 µg of plasmid and 4 × 10^5^ primary hepatocytes [41]. There have been no studies on plasmid delivery into hepatoblast cells or the testing of delivery protocols for cells derived from viral delivery methods [31,32]. Viral delivery methods, particularly AAV-based methods, are widely used in gene therapy, and several drugs employing these methods have already received FDA approval. Therefore, an important step in this work was to demonstrate the applicability of these viral vectors in the cellular models we created. Previous studies have shown that certain rAAV serotypes, such as rAAV7, 8, 9, and 3b, exhibit tropism for hepatocytes [40,42] The only published work comparing rAAV serotypes (rAAV2, rAAV3b, rAAV5, rAAV8, rAAVLK03, rAAVNP59, and rAAV2) in hiPSC-derived 3D hepatic organoids shows that rAAV2 is the most effective [36]. In our study, AAV2 also demonstrated the best results, with the reporter gene delivery efficiency reaching 94%, among the AAV serotypes evaluated (2/2, 2/5, 2/6, and 2/9). In our opinion, this is due to the presence of heparin sulfate proteoglycan (HSPG), the primary receptor for AAV binding, as well as co-receptors such as FGFR1, αVβ5, HGFR, and laminin receptors (LRs), which are widely expressed across various cell types, including hepatocytes [43,44,45] However, the full spectrum of receptors expressed on these cells remains incompletely characterized, underscoring the rationale for testing multiple serotypes.

## 5. Conclusions

We have optimized a protocol for differentiating human-induced pluripotent stem cells (hiPSCs) into liver progenitor cells (LPCs) in both two-dimensional (2D) and three-dimensional (3D) cultures, along with methods for transgene delivery in liver progenitors. These cell models have potential applications in the study of intercellular interactions to drive further differentiation and establish 3D cell structures (organoids). These cell lines are suitable for transfection and transduction, making them valuable for personalized therapy, such as genome editing, regenerative medicine, and disease modeling studies.

## Figures and Tables

**Table 1:** List of used antibodies.

Antibodies	Source	Identifier
Rabbit anti-SOX17	Abcam, UK	Cat#ab224637; RRID: AB_2801385
Rabbit anti-HNF1A	Abclonal, USA	Cat#A20865; RRID: AB_2728751
Rabbit anti-FOXA2	Abcam, UK	Cat#ab108422; RRID:AB_11157157
Mouse Anti-Alpha-Fetoprotein (AFP)	Abclonal, USA	Cat#A17898; RRID:AB_2861748
Rabbit Anti-Albumin	Abcam, UK	Cat#ab106582;RRID:AB_10888110
Rabbit Anti-Cytokeratin 18	Abcam, UK	Cat#ab133263; RRID:AB_11155892
Rabbit Anti-Cytokeratin 7	Abcam, UK	Cat#ab181598; RRID:AB_2783822
Anti-CD271 (NGF Receptor)-PE	Invitrogen, USA	Cat#12-9400-42; RRID:AB_2572710
a-SMA, alpha smooth muscle Actin Rabbit mAb	Abclonal, USA	Cat#A17910; RRID:AB_2861755
ZO-1	Abcam, UK	Cat#ab216880; RRID:AB_2909434
Goat anti-Mouse IgG (H + L), Alexa Fluor 594	Thermo Fisher Scientific, USA	Cat#A-11032; RRID:AB_2534091
Goat anti-Rabbit IgG (H + L), Alexa Fluor 594	Thermo Fisher Scientific, USA	Cat#A-11037; RRID:AB_2534095
Goat anti-Rat IgG (H + L), Alexa Fluor 488	Abcam, UK	Cat#ab150113; RRID:AB_2576208
Anti-Rabbit IgG H&L, Alexa Fluor 488	Abcam, UK	Cat#ab150077; RRID:AB_2630356

**Table 2:** List of hepatocyte cell lines and parameters for Neon protocol electroporation.

Cell Line	Neon Protocol Electroporation
ChangX-31	1050 V−50 ms—1 pulse
HepG2	1200 V−20 ms—3 pulse
SK-Hep-1	870 V−35 ms—2 pulse
H-4-II-E	1600 V−20 ms—1 pulse 1300 V−30 ms—1 pulse

Final transfection protocol using the Neon transfection system is as follows: 1200 V/20 ms/3 pulse with 2 μg/μL of AAV-CMV-GFP plasmid.

**Table 3:** List reagent of directed differentiation hiPSCS into hepatoblast cells protocols.

Publications	S. Altmaier et al. [1]		Y. Ao et al. [2]		X. Gao et al. [29]		N. Graffman et al. [24]		C. Du et al. [26]		Present Article	
Definitive endoderm												
Medium	MCDB131		RPMI		RPMI 1640 B27 supplement minus insulin		RPMI 1640 B27 supplement minus insulin		RPMI 1640 1% B27 without vitamin A		RPMI 1640 1% B27 without vitamin A	
Small molecules	Day 1	0.5% BSA 1.5 g/L NaHCO3 10 mM Glucose 1% Glutamax 0.1% Pen/Strep 100 ng/mL Activin A 3 µM CHIR99021	Day 1	100 ng/mL Activin A 25 ng/mL Wnt 3a	Day 1	3 μM CHIR99021	Day 1	1% Glutamax 1% Pen/Strep 100 ng/mL Activin A 2.5 μM CHIR99021	Day 1	3 μM CHIR99021	Day 1	1% Glutamax 1% sodium pyruvate 100 ng/mL Activin A 3 μM CHIR99021
	Day 2–3	0.5% BSA 1.5 g/L NaHCO3 10 mM Glucose, 1% Glutamax 0.1% Pen/Strep 100 ng/mL Activin A	Day 2–5	100 ng/mL Activin A 10 ng/mL bFGF	Day 2	-	Day 2–5	1% Glutamax 1% Pen/Strep 100 ng/mL Activin A	Day 2		Day 2–3	1% Glutamax 1% sodium pyruvate 100 ng/mL Activin A 10 ng/mL FGFb
Hepatocyte progenitors and specification (hepatoblast cells)												
	DMEM/F12		SFD		DMEM		Knockout DMEM		DMEM/F12 1% B27 Serum-Free-Supplement		RPMI1640 1% B27 without vitamin A	
Small molecules	Day 4–9	10% KOSR 1% Glutamax 1% Non-essential amino acids (NEAAs) 1% Pen/Strep 1% DMSO	Day 6 –8	10 ng/mL bFGF 50 ng/mL bone morphogenetic protein 4 (BMP4) 10 ng/mL epidermal growth factor (EGF) 100 ng/mL hepatic growth factor (HGF)	Day 3–8	1% DMSO 20% knockout serum replacement 2 mM Glutamax 1× MEM non-essential amino acids 100 μM 2-mercaptoethanol	Day 6–10	20% Knockout serum replacement 0.5% Glutamax 1% P/S, 0.01% 2-Mercaptoethanol 1% DMSO	Day 3–8	1% KOSR 1% Glutamax 1% NEAA 0.5 µM A83-01 250 nM sodium butyrate 0.5% DMSO	Day 4–6	1% Glutamax 1% sodium pyruvate 50 ng/mL FGF-10 10 µM Retinoid acid 10 µM SB431542
											Day 7–9	1%Glutamax 1% sodium pyruvate 50 ng/mL FGF-10 10 µM BMP4

Reagents that are the same at the one stage are highlighted in red.

## Data Availability

The data will be made available at reasonable request.

## Supplementary Materials

The following supporting information can be downloaded at: https://www.mdpi.com/article/10.3390/biology14060586/s1, Supplementary S1. Characterisation P12L3-iPSC line (*.docx), Video S1: Activated state of Stellate-like cells, Supplementary File «*.cpproj»—the macros used for the segmentation analysis. Supplementary S2 Figure S1: Immunofluorescence staining of definitive endoderm cells, liver progenitor cells (LPCs), and 3D liver organoids; Supplementary S2 Figure S2: Negative controls for immunocytochemical staining of liver progenitor cells (LPCs); Supplementary S2 Figure S3: Characterization of three-dimensional hepatic organoids.
