# Supplementary material for: The Optimization of a Protocol for the Directed Differentiation of Induced Pluripotent Stem Cells into Liver Progenitor Cells and the Delivery of Transgenes
Source: Biology (Basel). 2025 May 22;14(6):586. doi: 10.3390/biology14060586 (PMC12189164; doi:10.3390/biology14060586)
Supplement: Supplementary file 1 [file biology-14-00586-s001.zip › S1/P12L3, P16L4, P17L16 ipsc supl.pdf]

In present work using five iPSC-cell lines, two of them has been published. hiPSC lines P17L16 and P16L4 submitted to the journal for publication. You can find more detailed information by following links https://hpscreg.eu/cell-line/RCMGi016-A and https://hpscreg.eu/cell-line/RCMGi015-A.

P12L3 – iPSC cell lined obtained from fibroblasts 25-years old healthy woman. In supplementary present characterization iPSC line more detailed information present in dashboard https://hpscreg.eu/user/cellline/edit/RCMGi017-A.

1. Results

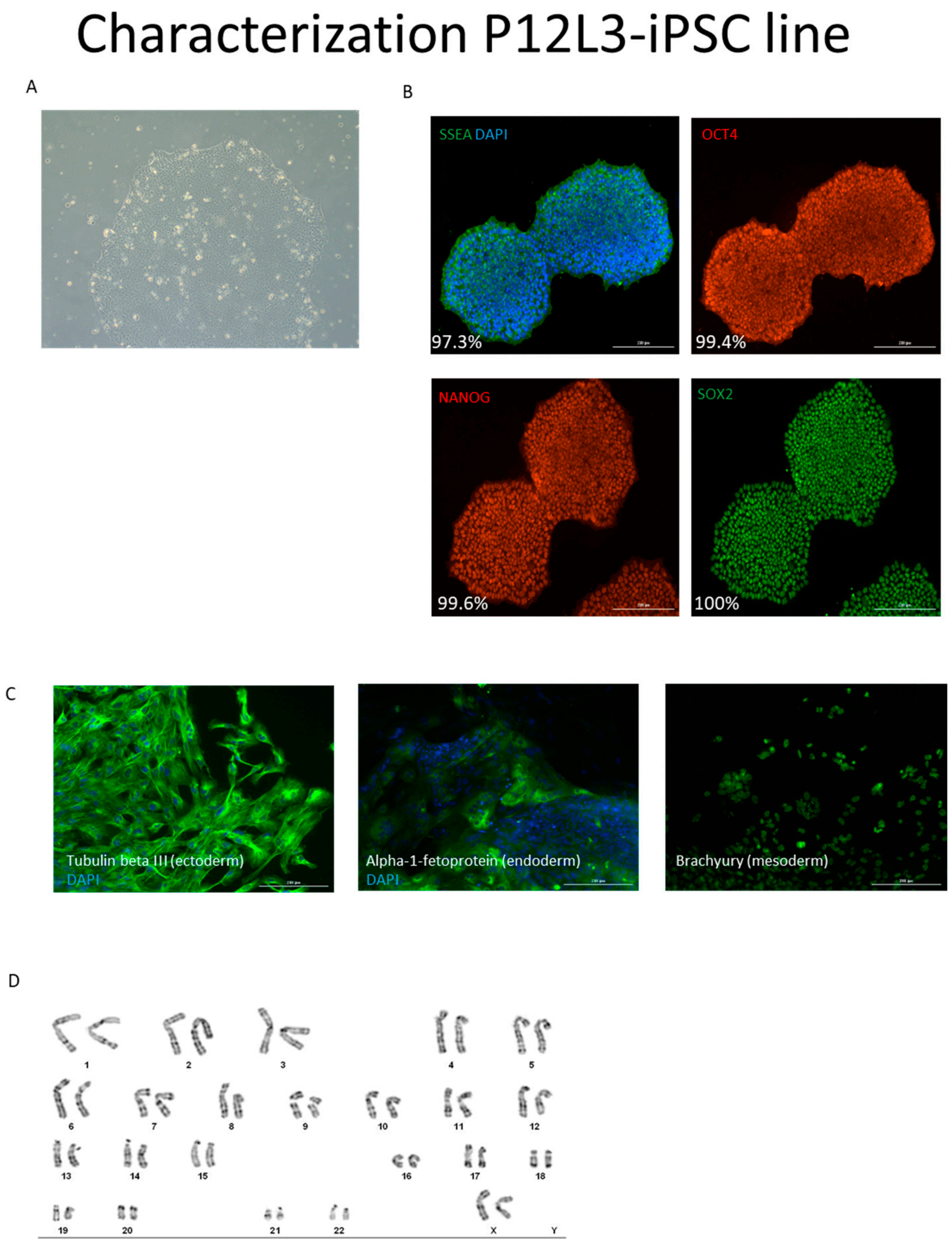

Supplementary Figure. A-- phase-contrast microscopy of iPSCs morphology, B- Immunocytochemistry of iPSCs colonies on pluripotent morphology. C-- Spontaneous differentiation iPSCs into three germ layers, D- karyotyping. Scale bar 200µM.

STR analysis

Marker	Fb-P12L3		iPSC-P12L3	
D21S1409	-	-	-	-
D13S252	283	295	283	295
D21S1442	357	369	-	-
D18S819	447	447	447	447
DXS1187	140	152	140	152
D21S1446	208	222	208	222
HPRT	280	280	280	280
D18S386	365	373	365	373
D13S305	447	457	447	457
DXS6803	135	147	135	147
D21S1435	-	-	181	185
D21S11	232	232	232	232
D13S634	-	-	387	387
D18S535	473	477	473	477
AMEL	107		107	107
D18S978	221	221	221	221
SRY	no	no	no	no
D13S800	295	299	295	299
D18S390	361	381	361	381
D13S628	458	462	458	462

2. Material and methods

2.1. Reprogramming and hiPSC maintenance

Human iPSCs were generated by reprogramming fibroblasts using a CytotuneiPS™-iPS 2.0 Sendai Reprogramming Kit (Life Technologies) according to the manufacture's recommendation. Generated iPSC lines were cultured onto Matrigel solution (Corning) coated plates with TeSR™-E8™ medium (Stemcell Technologies). The culture medium was changed daily. (Supl.Fig.A)

2.2. Karyotyping

The cultured cells at 12 passage and confluency ~ 70% were treated by 0,1 µg/mL demecolcine (Sigma) and then harvest by trypsinization, hypotonized 13 min in 0,075 M KCl at 37 °C and fixed using standard cytogenetic procedures. After that followed by the preparation of metaphase slides stained with DAPI and contrasted with 0,3 µg/mL Actinomycin (Serva). Finally, 15–20 metaphase images were analyzed according to ISCN 2020 nomenclature. . (Supl.Fig.D

2.3. Immunocytochemistry

IPSCs were fixed with 4% paraformaldehyde solution and blocking with 0.25% Triton X-100 in PBS and 1% bovine serum albumin (BSA) (Gibco), respectively. Fixed cells were incubated with primary antibodies at room temperature (RT) one hour and then with secondary antibodies at RT for 1 h in the dark, diluted in 1% BSA, respectively. Nuclei was stained with DAPI (Abcam) at RT for 10 min (Table S1). Fluorescent images were captures with Lionheart FX Automated

Microscope (BioTek). Amount of positive cell were analyzed by open-source software CellProfiler 3.0.0 (Supl.Fig.B).

2.4. EB-formation

For embryoid body formation the iPSCs after the passage 10 were detached using Versen solution, then dissociated into single cell suspension and transferred to ultra-low attachment plate with E8 medium with 5 mkM Y27632. On day 4, the medium was changed with new medium consisting of 1:1 of TeSR-E8 and ES medium (Advanced DMEM/F12 (Gibco), 20% KOSR (Gibco), 1% GlutaMAX, 1% MEM NEAA, 1% penicillin/streptomycin). On day 7, the medium was changed with EB medium consisting of 1⁄2 vol of ES medium and 1⁄2 vol of fibroblasts medium without FBS. Thereafter, the medium was renewed every 2–3 days with a stepwise increase in the percentage of FBS from 1 to 10%. On day 28, EBs were transferred onto gelatin-coated plates in EB medium with 10% FBS. On next step, EBs were cultured during 14 days, and after all the expression of markers was verified by immunocytochemical staining (Suppl.Table S1), (Supl.Fig.C)

Supplementary Table S1. Antibodies used for immunocytochemistry assay

	Antibody name	Dilution	Company Cat # and RRID
Pluripotency Markers	SSEA4 Monoclonal Antibody (MC813-70), Mouse	1:100	Thermo Fisher Scientific, Cat #: 41-4000, RRID:AB_2533506
	Anti-Oct4 antibody, Rabbit	1:100	Abcam, Cat #: ab18976, RRID:AB_444714
	Nanog Polyclonal Antibody, Rabbit	1:100	Thermo Fisher Scientific, Cat #: PA1-097X, RRID:AB_2539868
	Anti-SOX2 antibody [9-9-3], Mouse	1:200	Abcam, Cat #: ab79351, RRID:AB_10710406
Differentiation Markers	Anti-beta III Tubulin antibody [2G10], Mouse	1:300	Abcam, Cat # ab131205, RRID:AB_2256751
	Anti-alpha 1 Fetoprotein antibody [AFP-01], Mouse	1:200	Abcam, Cat #: ab3980, RRID:AB_304203
	Brachyury Monoclonal Antibody (X1AO2), Mouse	1:100	Thermo Fisher Scientific, Cat# 14-9770-82, RRID: AB_2573016
Secondary antibodies	Goat Anti-Rabbit IgG H&L (Alexa Fluor® 647)	1:200	Abcam, Cat # 150079, RRID:AB_2722623
	Goat Anti-Mouse IgG H&L (Alexa Fluor® 488)	1:200	Abcam, Cat # ab150113, RRID:AB_2576208
